# Genome-wide identification of the *CmHDZ* gene family in Chinese chestnut (*Castanea mollissima*) and analysis of their expression patterns under different types of abiotic stress

**DOI:** 10.3389/fpls.2026.1778593

**Published:** 2026-04-30

**Authors:** Xiurong Xu, Shiming Cheng

**Affiliations:** Zhejiang Academy of Forestry, Hangzhou, Zhejiang, China

**Keywords:** abiotic stress, Chinese chestnut, CmHDZ gene family, expression analysis, identification

## Abstract

The members of the homologous homeodomain-leucine zipper (HDZ) family of transcription factors play important roles in the ability of plants to cope with adverse conditions. In this study, we analyzed the genome of the Chinese chestnut (*Castanea mollissima*) to identify the *CmHDZ* genes involved in stress response. In total, 25 *CmHDZ* genes were identified, of which 24 were distributed across 12 chromosomes. Evolutionary analysis indicated that the 25 *CmHDZ* genes were divided into four subfamilies: HDZ I, II, III, and IV, comprising 13, 7, 4, and 1 members, respectively. All CmHDZ protein sequences contained highly conserved homeodomains (motif1). Subcellular localization prediction indicated that most of the CmHDZ members were localized to the nucleus, except for CmHDZ13, which was localized to the chloroplast. Further experiments confirmed the nuclear localization of CmHDZ15. Collinearity analysis revealed that 12 *CmHDZ* members formed eight pairs of homologous genes. Chinese chestnut shared the least number of gene pairs with rice and maize (10 and 9, respectively) and the highest number with *Arabidopsis thaliana* (up to 34 pairs). *Cis*-acting element prediction analysis indicated that the promoters of all 25 *CmHDZ* genes contained hormone and stress response elements. Moreover, the tissue-specific expression patterns of the *CmHDZ* genes varied under different types of stress. In particular, the *CmHDZ15* expression level changed significantly in response to shading, low-temperature, and high-temperature treatments. The quantitative analysis results confirmed the reliability of the transcriptome data. This study provides a genetic resource for screening resistance genes and for the molecular breeding of Chinese chestnuts.

## 1 Introduction

Transcription factors are the frontline defense molecules that plants use to resist various types of biological and abiotic stress, playing an important regulatory role in the resistance to adverse environmental stressors (Javed et al., 2020). The plant-specific HDZ family of transcription factors, which all contain a homeodomain and a leucine zipper (LZ), are responsible for regulating plant growth and development, hormone responses, and responses to adverse environmental stressors (Yang et al., 2023). The homeodomain, which is 60–61 amino acids in size, has the function of binding specifically to target DNA sequences (Yang et al., 2023; Salomone et al., 2024). The conserved domain of the LZ promotes the dimerization of the transcription factor monomers, which facilitates the specific binding of the homeodomain to the target DNA and thereby the regulation of target gene expression (Li et al., 2022). The HDZ transcription factors can be divided into four subfamilies (HDZ I through IV) on the basis of their conserved amino acid sequences, gene structures, and protein functions. The members of the HDZ I subfamily only possess the homeodomain and LZ motif, whereas those in the HDZ II subfamily also contain the characteristic CPSCE (Cys-Pro-Ser-Cys-Glu) and conserved N-terminal domains (Sharif et al., 2020). By contrast, the members of HDZ III and IV contain the StAR-related lipid transfer (START) domain and the START-associated domain (SAD), with the HDZ III members additionally possessing the MEKHLA (which stands for mostly eukaryotic known high-homology lipid-transfer associated) domain (Schrick et al., 2004; Mukherjee and Bürglin, 2006).

Owing to the structural differences among these four subfamilies, they also differ from one another in their expression patterns and biological regulatory functions. Studies have shown that HDZ I members play important roles in regulating the abiotic stress responses of plants and maintaining plant growth and development under adverse conditions. For example, the thale cress (*Arabidopsis thaliana*) contains 17 HDZ I subfamily members (Ariel et al., 2007), among which AtHB6 may act as a key regulatory factor in the abscisic acid (ABA) signaling pathway by interacting with the protein phosphatase 2C (PP2C)-encoding *ABI1* gene (Himmelbach et al., 2002). Moreover, AtHB7 and AtHB12 play important roles as negative regulators of ABA signal transduction in the plant response to drought and can interact with PP2C proteins to positively regulate the expression of *PP2C* genes (Valdés et al., 2012). A member of the HDZ I subfamily in cucumber (*Cucumis sativus* L.) was found to play an important role in mitigating NaCl and drought stress (Sharif et al., 2020). As observed in another study, the *MdHB-7* gene (encoding an HDZ I subfamily member) was expressed at different levels in different tissues of the apple plant (*Malus domestica* Borkh.) and its overexpression enhanced the drought tolerance of the apple tree (Zhao et al., 2020). Matsumoto et al. (2014) found that after applying drying, salt, cold, and ABA treatments to barley (*Hordeum vulgare*), the expression level of the HDZ I subfamily transcription factor HvHOX22 in seedlings was significantly increased. Maize (*Zea mays*) ZmHDZ9 plays a crucial role in regulating ABA and lignin production, and overexpression of the *ZmHDZ9* gene can significantly enhance the drought resistance of the plant (Jiao et al., 2024). Studies have also shown that multiple members of the *A. thaliana* HDZ I subfamily (e.g., AtHB1, AtHB3, AtHB13, AtHB20, and AtHB23) are involved in regulating the development of cotyledons and leaves (Aoyama et al., 1995; Henriksson et al., 2005; Mattsson et al., 2003).

The members of the HDZ II subfamily mainly participate in the photoreaction pathway and hormone signal transduction (Sharif et al., 2021). The *cis*-elements of the HDZ II subfamily genes in the catnip plant (*Nepeta cataria* L.) have been found to frequently contain light, ABA, and methyl jasmonate response elements, jointly regulating growth and secondary metabolism in the plant. Of the 10 HDZ II subfamily members in *A. thaliana*, five (AtHB2, AtHAT1, AtHAT2, AtHB4, and AtHAT3) have been shown to be involved in phototropism and auxin response, inducing the shade avoidance response of the plant according to the light quality conditions (Ciarbelli et al., 2008). Additionally, high expression of the growth hormone-induced gene *AtHAT2* results in tissue elongation in *A. thaliana* (Morelli and Ruberti, 2002). Furthermore, *HaHB10*, the homologous gene of *AtHB2* in the sunflower (*Helianthus annuus*) (Dezar et al., 2011), has been confirmed to have photoperiod-dependent effects, with its overexpression resulting in the plant exhibiting an early-flowering phenotype.

The members of the HDZ III subfamily are involved in embryogenesis (Roodbarkelari and Groot, 2017), apical meristem development, and the polar transport of auxin during plant development (Prigge et al., 2005). *A. thaliana* has five HDZ III subfamily genes; namely *AtHB8*, *PHV/AtHB9*, *PHB/AtHB14*, *CNA/AtHB15*, and *REV/IFL1*. The proteins encoded by *REV*, *PHB*, and *PHV* have functional redundancy, playing key roles in the development of the apical meristem of *Arabidopsis* (Elhiti and Stasolla, 2009). Because several members of the HDZ III class are known to be related to the polar transport of auxin, the regulation of these morphogenesis events may be caused by changes in auxin flow (Baima et al., 2001). Moreover, two members of this subfamily (AtHB8 and AtHB15) are important regulators of vascular development in plants (Kim et al., 2005). In the poplar plant (*Populus* L.), overexpression of the HDZ III member *PCN* negatively regulates the development of secondary vascular tissues (Du et al., 2011).

The members of the HDZ IV subfamily mainly participate in the development and maintenance of the epidermal cell layer and thus tend to be highly expressed in these cells (Peterson et al., 2013). They are also involved in the accumulation of anthocyanins and epidermal cell differentiation (Gu et al., 2024). The concentration of the HDZ IV protein woolly (wo) in the tomato plant (*Solanum lycopersicum* L.) directly determines the division potential of the epidermal hair cells (Wu et al., 2022). The HDZ IV members in *A. thaliana* are AtGL2, AtANL2, AtML1, AtPDF2, and AtHDG1–AtHDG12, among which AtGL2, AtHDG11, and AtHDG12 have been shown to regulate epidermal hair development (Szymanski et al., 1998; Wu et al., 2022). In *A. thaliana*, the initiation of epidermal hairs is regulated by the MYB–bHLH–WD40 complex formed by the R2R3–MYB transcription factors GL1 and WER, the bHLH transcription factor GL3/EGL3, and the WD40 transcription factor TTG1. This complex can activate the expression of the downstream HDZ IV subfamily gene *GL2*, thereby promoting the function of its encoded protein (Nakamura et al., 2006). Simultaneously, knockout of the *AtML1* and *AtPDF2* genes in *A. thaliana* leads to severe defects in the differentiation of epidermal bud cells, causing their failure to survive after germination, indicating that these two transcription factors have important roles in regulating the differentiation of epidermal cells (Elhiti and Stasolla, 2009). Studies have also found that AtANL2 affects the accumulation of anthocyanins in the lower layer of the leaf epidermis and participates in the regulation of primary root cell development (Abe et al., 2003; Ishida et al., 2008). In the sweet wormwood (*Artemisia annua*), overexpression of the HDZ IV subfamily genes *AaHD8* and *AaHD1* promotes the initiation of glandular hairs, and AaHD8 can form a complex with the MYB-type transcription factor AaMIX1, jointly activating the expression of AaHD1 and thereby promoting the development of glandular hairs and synthesis of artemisinin (Yan et al., 2017; Shi et al., 2018).

The Chinese chestnut (*Castanea mollissima*; genus: *Castanea*; family: Fagaceae) is an important woody crop that is native to China and other Asian countries (Ji et al., 2018). The species is widely distributed in barren and arid mountainous areas as well as sandy wastelands and is therefore exposed to various types of abiotic stress (including drought, cold, and heat) throughout the year, which seriously affect its growth, development, and yield (Zhang et al., 2024). Therefore, identifying the main stress resistance genes of *C. mollissima* and clarifying their regulatory mechanisms are of great significance for identifying Chinese chestnut germplasm resources and guiding associated cultivation and management strategies (Wang et al., 2024). Although several transcription factors related to stress resistance have been identified in *C. mollissima* (Wang et al., 2024; Xu et al., 2025, 2026), research on the HDZ family remains lacking.

In recent years, members of the HDZ family have been successfully identified in multiple species via functional analysis studies. For instance, the functions of 48 HDZ family members in *A. thaliana* have mostly been clarified (Mukherjee et al., 2009), and 55, 78, and 63 *HDZ* genes have been identified in *Z. mays* (Zhao et al., 2011), moso bamboo (*Phyllostachys edulis*) (Gao et al., 2021), and *Populus* spp (Guo et al., 2021), respectively. However, no studies to date have reported on the *CmHDZ* gene family in *C. mollissima*. Therefore, we performed a genome-wide analysis to identify the *CmHDZ* gene family of *C. mollissima* on the basis of its genome data and analyzed related bioinformatics data with the aim of providing a genetic resource that can support both further research on the stress resistance mechanisms of the species and the development of improved varieties.

## 2 Materials and methods

### 2.1 Acquisition of *HDZ* gene sequences in *C. mollissima*

Using HMMER 3.0 software, a Hidden Markov Model was constructed on the basis of the known HDZ protein sequences of *A. thaliana* and rice (*Oryza sativa*) and used to search for all potential *HDZ* genes in the protein-coding sequences of the *C. mollissima* genome. The potential CmHDZ protein sequences obtained were compared with the reference *Arabidopsis* HDZ sequences using Blastp (version: ncbi-blast-2.10.1+), with the e-value set at 1 × e^–5^. Sequences that matched were considered candidates of the HDZ family. Pfamscan v1.6 software and the Pfam Av33.1 database were used to annotate the domains of the candidates, with sequences containing PF00046 and PF02183 domains identified as CmHDZ family members. The subcellular localization of the CmHDZ family members was predicted using Softberry (http://www.softberry.com/). A physical map of the CmHDZ gene family on the chromosome was drawn using MG2C (http://mg2c.iask.in/mg2c_v2.1/).

### 2.2 Construction of a phylogenetic tree of the CmHDZ protein sequences

A phylogenetic tree of the CmHDZ and *Arabidopsis* HDZ protein sequences was constructed using the neighbor-joining method (Su et al., 2023). In brief, multiple sequence alignment was performed using MAFFT v7.427, and the phylogenetic tree was then drawn using MEGA10 software. For tree construction, the p-distance model was applied, a partial deletion method was used to handle missing data, the cutoff was set at 50%, and 1000 bootstrap samples were used to test the reliability of the branches of the calculated tree. The phylogenetic tree was annotated using iTOL v6 (https://itol.embl.de/).

### 2.3 Analysis of the structure and conserved motifs of the *CmHDZ* genes

The distribution of introns and exons in the *CmHDZ* gene sequences was analyzed using the Gene Structure Display Server (http://gsds.gao-lab.org/). The conserved motifs of the genes were analyzed using MEME v5.0.5, with the number of conserved motifs set to 15.

### 2.4 Analysis of *cis*-acting elements in the promoter regions of the *CmHDZ* genes

The 2 kb region upstream of the promoter sequence of each *CmHDZ* gene was extracted, and the *cis*-acting elements in the promoter regions were predicted and analyzed using the PlantCARE database (http://bioinformatics.psb.ugent.be/webtools/plant-care/html/).

### 2.5 Collinearity analysis of *C. mollissima*

The *C. mollissima* genome was scanned using MCScanX software. The gene duplication types of the entire genome were analyzed on the basis of the collinearity analysis results using the DupGenFinder program, and those of the *CmHDZ* genes were obtained (Qiao et al., 2019). The duplication types of the *CmHDZ* gene family members were obtained in the same way for eight other species (*A. thaliana*, *O. sativa*, *Vitis vinifera*, *Z. mays*, *Quercus dentata*, *Castanopsis tibetana*, *Castanea dentata*, and *Castanea crenata*). Finally, the nonsynonymous/synonymous substitution rates of the *CmHDZ* gene pairs with different duplication types were calculated using TBtools software (Chen et al., 2023).

### 2.6 Analysis of the *CmHDZ* gene expression patterns under abiotic stress

FASTQ files of the transcriptome with the Genome Sequence Archive identifier CRA022911 were obtained from the Chinese National Genome Database (https://ngdc.cncb.ac.cn/gsa). These data were from a study that explored the transcriptomes of 2-year-old *C. mollissima* cv. ‘Yanbao’ seedlings under 10 days of different low-light stress (i.e., 0%, 50%, 75%, and 95% shading intensities).

FASTQ files of the transcriptome with the identifier PRJNA1166987 were obtained from the NCBI database (https://www.ncbi.nlm.nih.gov/). The data were from a study that explored the transcriptome of *C. mollissima* cv. ‘Yanshan Zao Feng’ seedlings under low-temperature (D0h, D5h, D10h, and D15h) and high-temperature stress (G0h, G4h, G8h, and G12h). Here, D0h, D5h, D10h, and D15h represent treatments at –15 °C for 0, 5, 10, and 15 h, respectively, whereas G0h, G4h, G8h, and G12h represent treatments at 45 °C for 0, 4, 8, and 12 h, respectively.

FASTQ files of the transcriptome with the identifier PRJNA731244 were obtained from the NCBI database (https://www.ncbi.nlm.nih.gov/). The data were from our previous study that explored the transcriptome of *C. mollissima* cv. ‘Yanshan Zao Feng’ under drought stress (0d, 10d, 20d, 30d, and 40d). Here, 0d, 10d, 20d, 30d, and 40d represent the treatment of the seedlings to continuous waterless conditions for 0, 10, 20, 30, and 40 days, respectively (Xu et al., 2025).

All transcriptome data were sequenced on the Illumina platform in paired-end mode, and the reads were aligned to the reference genome of *C. mollissima* cv. N11-1. Finally, the gene expression level was determined as a fragments per kilobase of transcript per million fragments mapped (FPKM) value.

### 2.7 Plant materials and qPCR validation

To investigate differences in the phenotypes, transcriptomes, and expression levels of chestnut leaves under different abiotic stress intensities, we used 2-year-old *C. mollissima* cv. ‘Maoban Hong’ seedlings exhibiting consistent growth potential under standard water and fertilizer conditions as the test material. The seedlings were subjected to shading treatment using black shading nets. Four shading intensities were used: 0%, 50%, 75%, and 95%. After 10 days of treatment, the third leaves from the top of the seedlings were collected, rapidly frozen in liquid nitrogen, and stored at –80 °C. The trees were then subjected to high-temperature treatment at 45 °C and leaf samples were collected after 4, 8, and 12 h. Additionally, trees were subjected to low-temperature treatment at –15 °C and leaf samples were collected after 5, 10, and 15 h. Trees grown at 25 °C were used as controls. Each treatment was replicated three times, with three chestnut seedlings per replicate. All collected samples were rapidly frozen in liquid nitrogen and stored at –80 °C before further use.

The quantitative real-time polymerase chain reaction (qPCR) was performed using the TB Green Premix Ex Taq kit (TaKaRa, Dalian, China). The relative expression levels of the *CmHDZ* genes at different time points were calculated using the 2^–ΔΔCt^ method (Livak and Schmittgen, 2001). The PCR thermal cycling conditions were as follows: an initial 95 °C for 300 s, followed by 40 cycles of 95 °C for 10 s and 60 °C for 30 s. Information on the specific primers used is shown in **Supplementary Table 1**. The *C. mollissima* actin gene was used as the reference gene.

### 2.8 Subcellular localization of CmHDZ15

The *Bsa*I restriction endonuclease was used for seamless cloning of the *CmHDZ15* open reading frame (ORF) into the pAN580 vector. The specific primer was designed with the recognition site of *Bsa*I (5′-GGTCTC-3′) incorporated to leverage its property of cutting outside the recognition site to generate specific sticky ends, thereby enabling directional ligation of the target fragment to the pAN580 vector. Using the seamless cloning technique, the *CmHDZ15* ORF sequence with the stop codons removed was ligated to pAN580, and green fluorescent protein (GFP) was fused to the N-terminus. The recombinant vectors were then introduced into tobacco protoplasts via polyethylene glycol-mediated transformation. GFP fluorescence signals were observed under 470 nm excitation light using a confocal laser scanning microscope to determine the subcellular localization of the CmHDZ15 protein.

## 3 Results

### 3.1 CmHDZ family members in *C. mollissima* and their physiocochemical properties

After searching the whole-genome data of *C. mollissima* (using MEGA7.0 for comparison with the genomes of *A. thaliana* and *O. sativa*) and removing redundant sequences, 25 *CmHDZ* genes (named *CmHDZ1*–*CmHDZ25*, respectively) were identified on the basis of their positions on the chromosomes (**Table 1**). Sequence analysis revealed that the predicted protein sequences encoded by the various *CmHDZ* genes varied greatly, with lengths ranging from 168 (CmHDZ11) to 843 (CmHDZ13) amino acids (corresponding to molecular weights of 19.69–92.19 kDa) and theoretical isoelectric points (pI) ranging from 4.71 (CmHDZ16) to 9.45 (CmHDZ21). The instability index ranged from 47.13 (CmHDZ21) to 78.21 (CmHDZ14), with an average value of 56.29. The average hydrophobicity values ranged from –1.115 (CmHDZ11) to –0.098 (CmHDZ13), indicating that these transcription factors were all hydrophilic proteins, albeit with varying degrees of hydrophilicity. Based on the hydrophobicity values, the most hydrophilic protein was CmHDZ11 and the least hydrophilic was CmHDZ13. This suggests that most of the CmHDZ proteins are unstable in nature. Subcellular localization prediction indicated that the majority of the CmHDZ members were located in the nucleus, except for CmHDZ13, which was located in the chloroplasts.

**Table 1:** Basic characteristics of the putative proteins encoded by the 25 *CmHDZ* genes.

Gene ID	Chromosome ID	Gene name	Number of amino acids	Molecular weight	Theoretical pI	Instability index	Aliphatic index	Grand average of hydropathicity	Subcellular localization
EVM0015907	Chr1	*CmHDZ1*	278	31722.45	5.80	59.37	68.42	-0.829	nucleus
EVM0016208	Chr1	*CmHDZ2*	287	32313.52	8.31	57.72	68.36	-0.824	nucleus
EVM0019525	Chr1	*CmHDZ3*	198	22875.61	6.92	56.90	66.06	-0.987	nucleus
EVM0032854	Chr1	*CmHDZ4*	275	31353.35	6.42	60.89	65.27	-0.800	nucleus
EVM0014166	Chr2	*CmHDZ5*	753	83648.74	5.84	54.56	74.09	-0.48	nucleus
EVM0025314	Chr2	*CmHDZ6*	839	91893.79	5.92	52.77	86.76	-0.133	nucleus
EVM0032379	Chr2	*CmHDZ7*	816	89774.08	6.36	48.60	87.63	-0.118	nucleus
EVM0028450	Chr3	*CmHDZ8*	321	35822.79	8.65	63.10	59.84	-1.017	nucleus
EVM0016424	Chr4	*CmHDZ9*	285	32347.33	5.52	60.11	63.37	-0.816	nucleus
EVM0017389	Chr4	*CmHDZ10*	295	33722.32	4.78	49.52	66.37	-0.811	nucleus
EVM0017508	Chr4	*CmHDZ11*	168	19694.75	4.98	53.56	66.13	-1.115	nucleus
EVM0024481	Chr5	*CmHDZ12*	243	27756.49	4.80	55.62	58.23	-1.009	nucleus
EVM0025058	Chr6	*CmHDZ13*	843	92195.93	5.96	50.90	87.39	-0.098	chloroplast
EVM0025544	Chr6	*CmHDZ14*	228	25676.48	8.61	78.21	72.28	-0.525	nucleus
EVM0028117	Chr7	*CmHDZ15*	322	36227.27	5.08	62.86	65.12	-0.757	nucleus
EVM0005201	Chr8	*CmHDZ16*	329	36994.73	4.71	53.29	70.21	-0.780	nucleus
EVM0015070	Chr8	*CmHDZ17*	202	23844.75	6.37	51.01	62.77	-0.998	nucleus
EVM0018144	Chr9	*CmHDZ18*	235	26656.01	7.57	51.77	66.00	-0.834	nucleus
EVM0003602	Chr10	*CmHDZ19*	355	39283.67	6.92	58.88	58.62	-0.810	nucleus
EVM0022981	Chr10	*CmHDZ20*	225	25948.28	7.69	49.33	63.73	-0.880	nucleus
EVM0023488	Chr11	*CmHDZ21*	219	25136.95	9.45	47.13	74.38	-0.921	nucleus
EVM0000545	Chr12	*CmHDZ22*	836	92039.46	6.00	47.38	86.12	-0.121	nucleus
EVM0006976	Chr12	*CmHDZ23*	220	25162.27	6.46	56.71	67.00	-0.892	nucleus
EVM0013297	Chr12	*CmHDZ24*	293	32999.21	7.60	64.44	66.21	-0.791	nucleus
EVM0020240	Contig1	*CmHDZ25*	319	35598.54	8.44	62.69	59.00	-1.006	nucleus

### 3.2 Chromosomal localization of the *CmHDZ* genes

**Figure 1:**
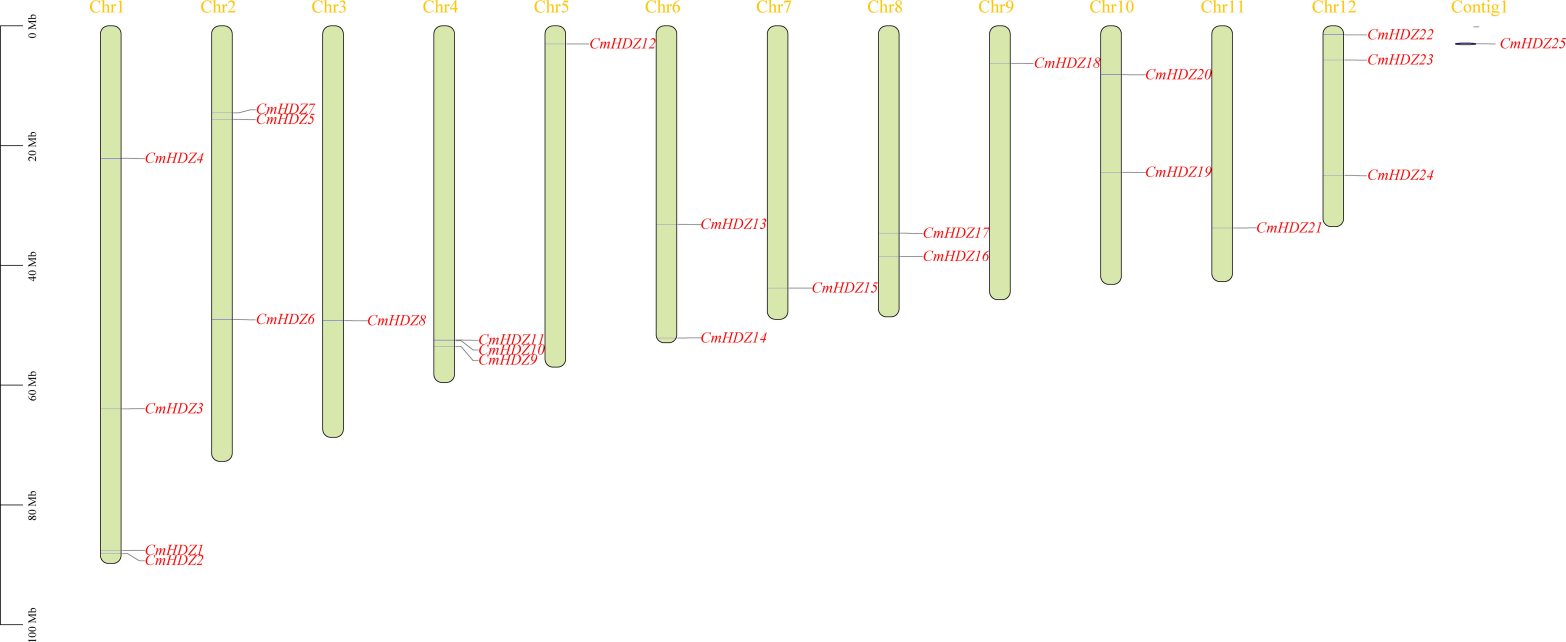
Distribution of the *CmHDZ* genes on the *Castanea mollissima* chromosomes. Vertical colored bars represent the chromosomes of *C. mollissima*. The gene name and number are shown at the right/left of each chromosome. The scale bar on the left represents the length of the chromosomes.

The chromosomal localization analysis showed that the 25 *CmHDZ* genes were unevenly distributed across the 12 chromosomes of *C. mollissima*, and *CmHDZ25* did not map to the chromosomes (**Figure 1**). Chromosome 1 contained the largest number of the genes, with four members. Chromosomes 2, 4, and 12 had three members each, chromosomes 6, 8, and 10 had two members each, and chromosomes 3, 5, 7, 9, and 11 had only one gene member each. Additionally, one homologous gene cluster comprising *CmHDZ10* and *CmHDZ11* was identified.

### 3.3 Phylogenetic tree of the CmHDZ protein family

**Figure 2:**
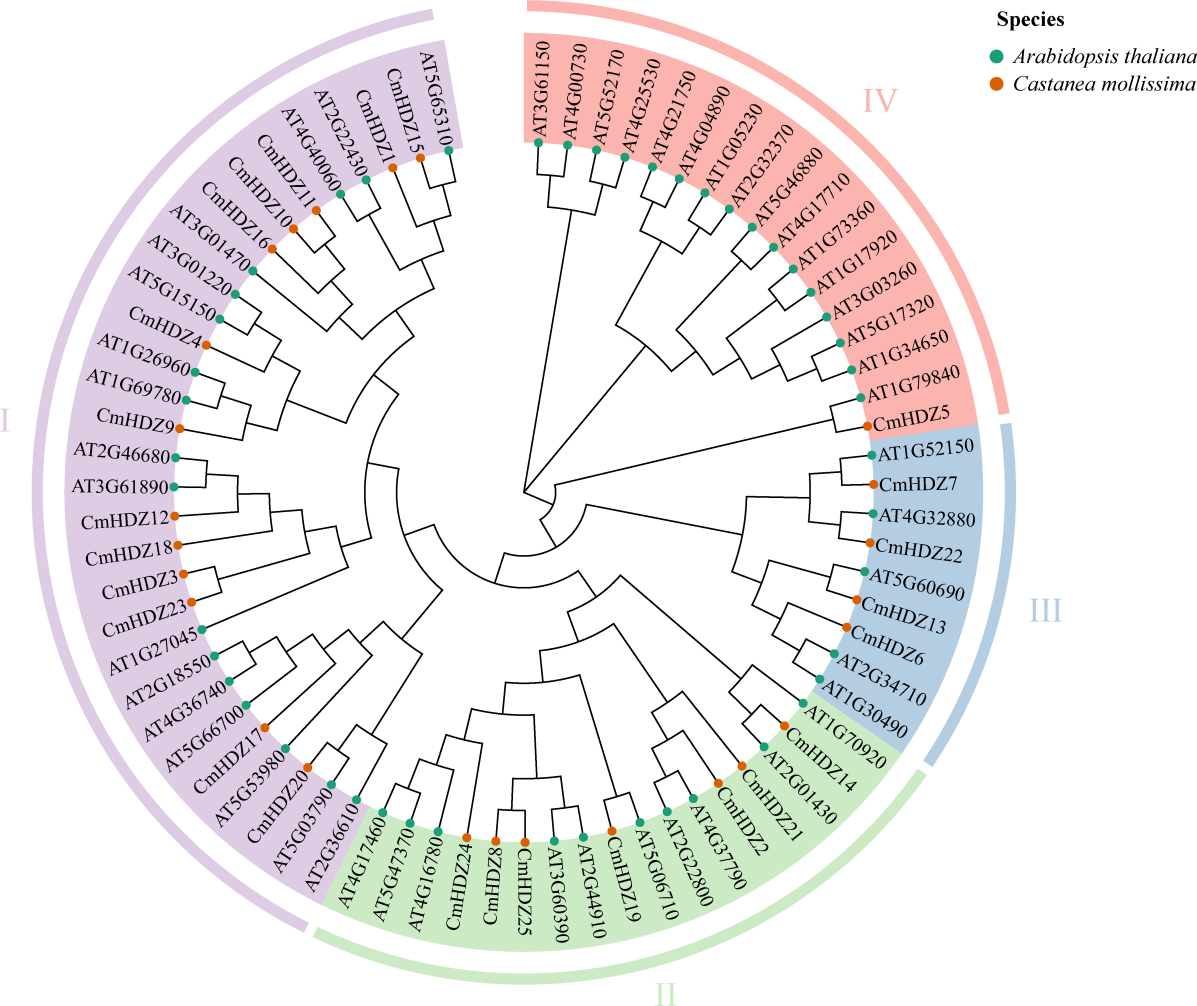
Phylogenetic tree based on the amino acid sequences. The different branch colors indicate different groups; the different colors of gene names indicate different orthogroups.

A phylogenetic tree was constructed using the HDZ protein sequences from *A. thaliana* and *C. mollissima*. The results showed that the classification of the *C. mollissima* genes was similar to those of *A. thaliana*. Similar to the AtHDZ proteins, the 25 CmHDZ proteins were divided into four subfamilies: HDZ I, II, III, and IV, each containing 13, 7, 4, and 1 members, respectively. Among all the HDZ subfamilies, HDZ I tends to contain the largest number of members, with 17 in *A. thaliana* and 13 in *C. mollissima*. The phylogenetic tree also showed that HDZ II, III, and IV were clustered together and more closely related to one another than to HDZ I. *AT1G79840* and *CmHDZ5*, *AT1G52150* and *CmHDZ7*, and *AT4G32880* and *CmHDZ22* were identified as homologous genes between *A. thaliana* and *C. mollissima* (**Figure 2**). None were identified between *C. mollissima* and *O. sativa.* The functions of these homologous genes in *A. thaliana* can provide a scientific basis for predicting the functions of the corresponding gene in *C. mollissima*. Notably, the HDZ IV subfamily of *A. thaliana* has a large number of members, whereas that of *C. mollissima* contains only one, which suggests that a gene reduction phenomenon has occurred during the evolution of the Chinese chestnut plant.

### 3.4 Conserved domains of the CmHDZ proteins and their gene structures

**Figure 3:**
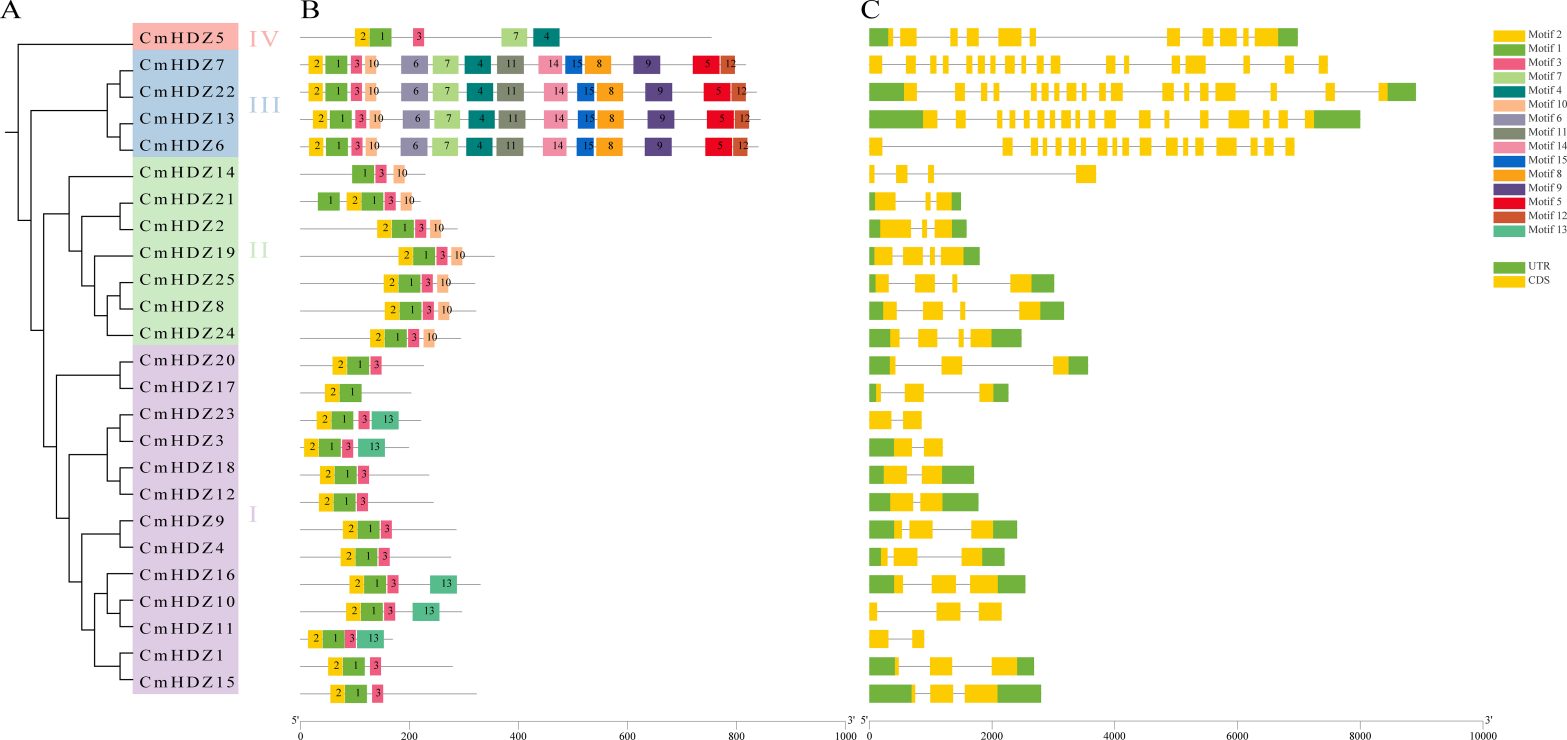
Motif and gene structure analysis. **(A)** Evolutionary tree of the CmHDZ family. **(B)** The 15 conserved motifs in CmHDZ proteins. Conserved motifs of the CmHDZ proteins were identified on the basis of the 25 full-length amino acid sequences using the online MEME program with the following parameters: maximum number of motifs, 15; maximum width, 100. The lengths and positions of the different motifs in the protein sequences are identified by the lengths and positions of the different color blocks. **(C)** Structure of the *CmHDZ* genes. The exons, introns, and untranslated regions (UTRs) are indicated by green rectangles, black lines, and yellow rectangles, respectively.

Protein family members with similar conserved domains generally have similar functions. To better understand the similarity and diversity of the protein domains in the CmHDZ members, their conserved motifs were analyzed using MEME v5.0.5 software (**Figure 3B**). The results showed that motifs 1, 2, and 3 were present in almost all the proteins, except CmHDZ14 (which did not contain motif 2) and CmHDZ17 (which did not contain motif 3). According to the NCBI Conserved Domain database (https://www.ncbi.nlm.nih.gov/Structure/cdd/wrpsb.cgi), motif 1 is a homologous domain and motif 3 is a homeobox-associated LZ. Together, these three structures constitute the conserved motifs of the *CmHDZ* gene family, which is consistent with the structural basis used to classify the CmHDZ proteins. Members of HDZ III had more motifs than those of the other three subfamilies. Motifs 4 and 7 were only present in HDZ III and IV, whereas motif 10 was only found in HDZ II and III and motif 13 in HDZ I. The number of introns varied among the subfamilies as follows (**Figure 3C**): 1–2 in HDZ I, 2–3 in HDZ II, 10 in HDZ III, and 17 in each of the members of HDZ IV. These results are consistent with the simpler structures of the genes in the HDZ I and II subfamilies of other species. Further comparative analysis revealed that the structural similarity was higher between genes clustered closely on the evolutionary tree.

### 3.5 *Cis*-acting elements in the promoters of the *CmHDZ* genes

**Figure 4:**
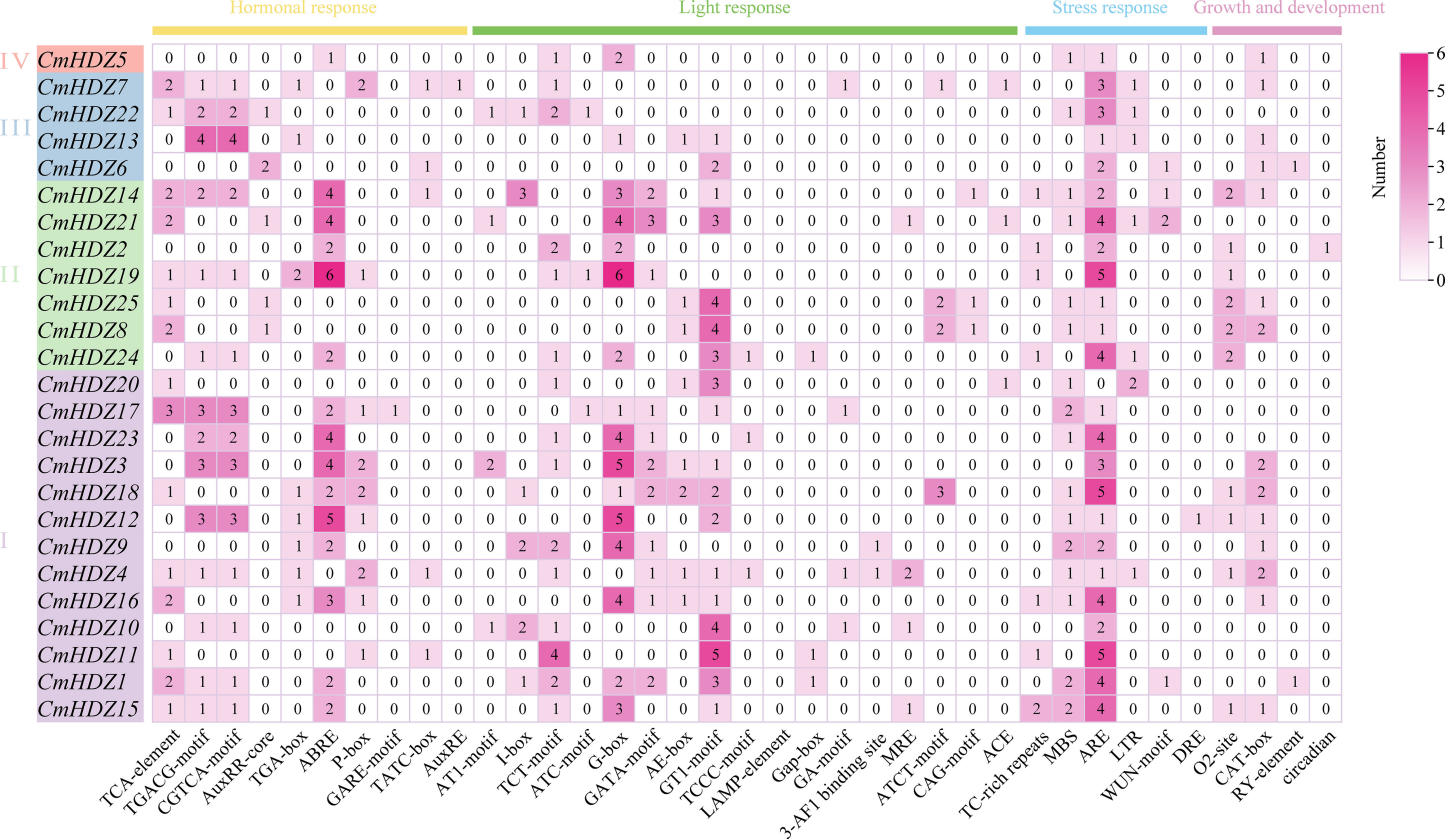
*Cis-*elements in the promoter regions of the *CmHDZ* gene*s.* The colored block with a number represents the *cis*-element number of the gene.

In total, 485 *cis*-acting elements were identified in the 2 kb region upstream of the *CmHDZ* genes (**Figure 4**, **Supplementary Table 2**). In addition to the basic promoter core elements (TATA and CAAT boxes), light-responsive elements (G and AT~TATA boxes), methyl jasmonate-responsive elements (CGTCA and TGACG motifs), ABA-responsive element (ABRE), and stress-responsive element (LTR) were also present. The antioxidant response element (ARE) was the most abundant, being present in all the *CmHDZ* genes except for *CmHDZ9* and *CmHDZ19*. The G-box and ABRE were the second most abundant elements. These findings indicate that the expression levels of the *CmHDZ* gene family may be affected by various factors, such as light, adverse environmental stress, hormones, and transcription factors, thereby participating in the regulation of *C. mollissima* growth and development processes.

### 3.6 Collinearity of the *CmHDZ* genes

**Figure 5:**
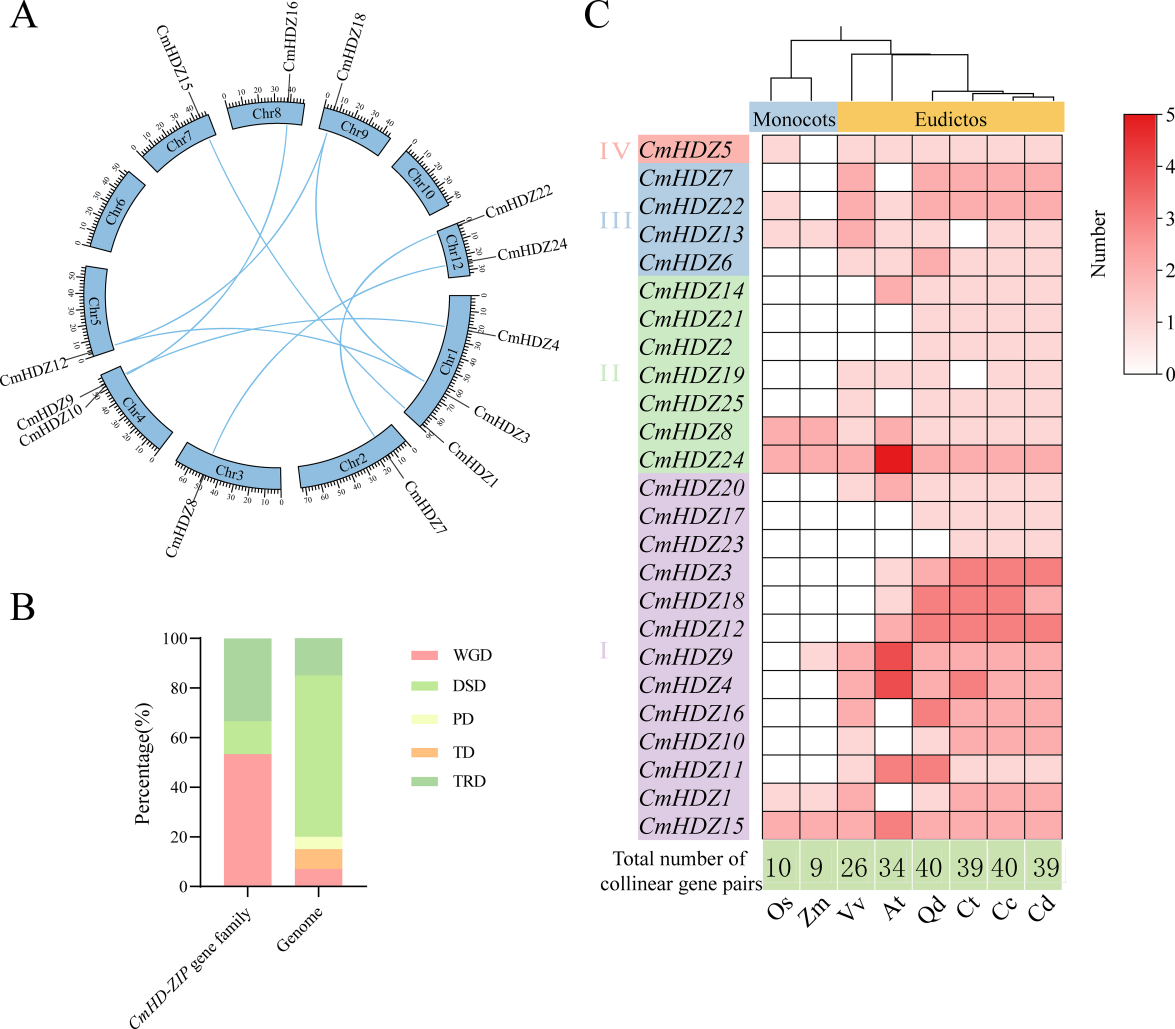
Collinearity and duplication types in the *CmHDZ* gene family. **(A)** Intragenomic collinearity of the *CmHDZ* genes. **(B)** Duplication types in the *CmHDZ* genes. TRD, transpositional duplication; DSD, dispersed duplication; PD, proximal duplication; TD, tandem duplication; WGD, whole-genome duplication. **(C)** Number of genetic combinations of *Castanea mollissima* with other species.

To elucidate the potential evolutionary mechanism of the *CmHDZ* genes, we studied the replication events of this family. The results showed that 12 of the 25 *CmHDZ* genes had duplicated segments, which were located on nine chromosomes and formed eight homologous gene pairs (**Figure 5A**). Paired collinear genes may have extremely similar functions. With regard to the gene replication types, both the proximal and tandem duplication types were completely lost from the entire *CmHDZ* genome, whereas the transpositional and whole-genome duplication types were markedly increased and the dispersed duplication proportion was substantially decreased (**Figure 5B**). Calculation of the nonsynonymous/synonymous substitution rates yielded Ka/Ks values of less than 0.3 for all eight gene pairs (**Supplementary Table 3**). A subsequent analysis of the collinearity between the *CmHDZ* genes and the *HDZ* genes of *A. thaliana*, *O. sativa*, *V. vinifera*, *Z. mays*, *Q. dentata*, *Castanopsis tibetana*, *Castanea dentata*, and *Castanea crenata* was performed and a heatmap was drawn. According to the results, *C. mollissima* shared the highest number of homologous genes (ranging from 39 to 40 pairs) with several plants belonging to the Fagaceae (*Q. dentata*, *Castanopsis tibetana*, *Castanea dentata*, and *Castanea crenata*), whereas it shared the fewest gene pairs with *O. sativa* (10 pairs) and *Z. mays* (9 pairs). The highest number of gene pairs shared was with *A. thaliana* (34 pairs).

### 3.7 qPCR validation of the transcriptome-based expression patterns

**Figure 6:**
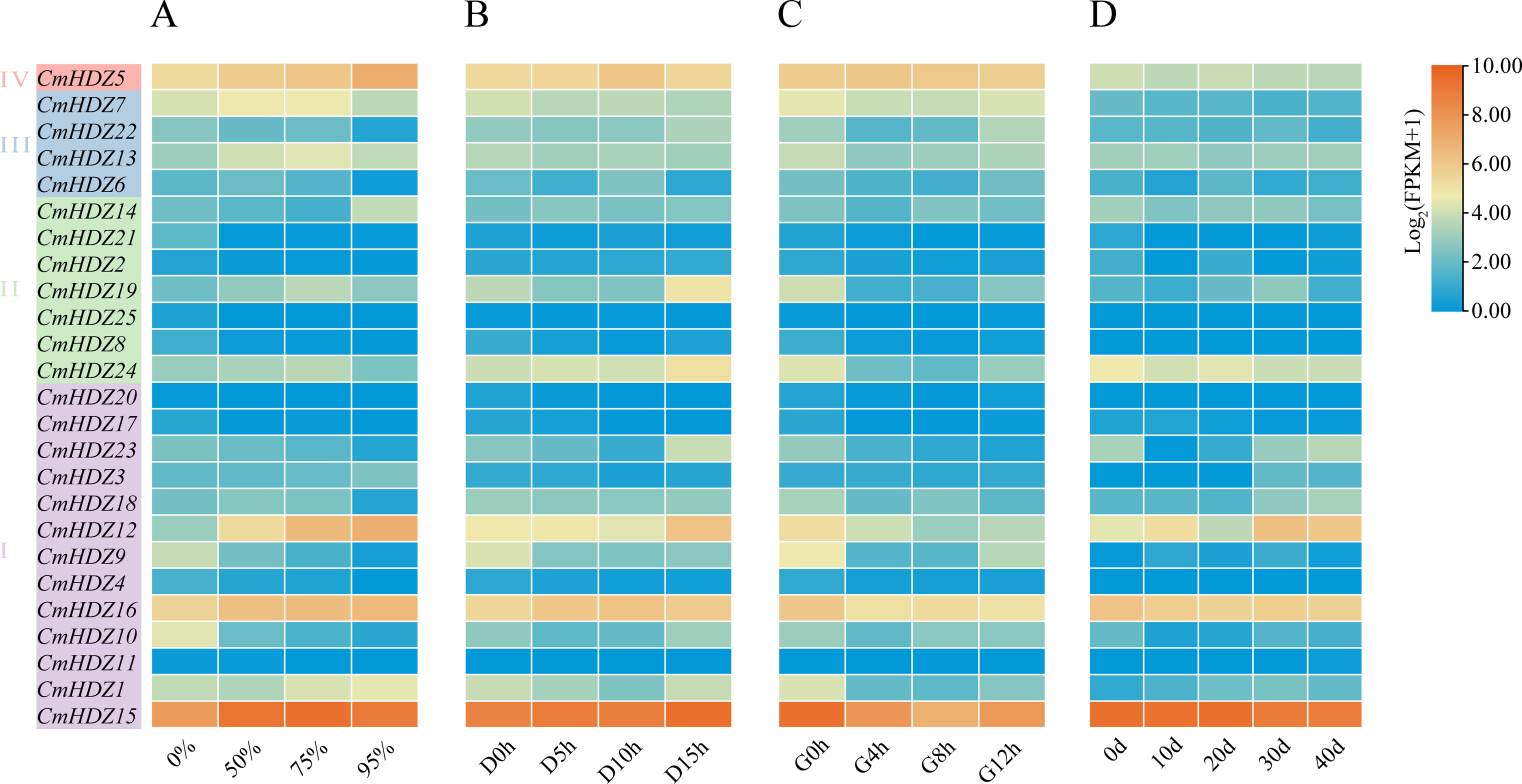
Expression pattern of the *CmHDZ* genes. **(A)** 0%, 50%, 75%, and 95% represent the different percentages of shading. **(B)** D0h, D5h, D10h, and D15h represent the results after low-temperature treatment for 0, 5, 10, and 15 h, respectively. **(C)** G0h, G4h, G8h, and G12h represent the results after high-temperature treatment for 0, 5, 10, and 15 h, respectively.

**Figure 7:**
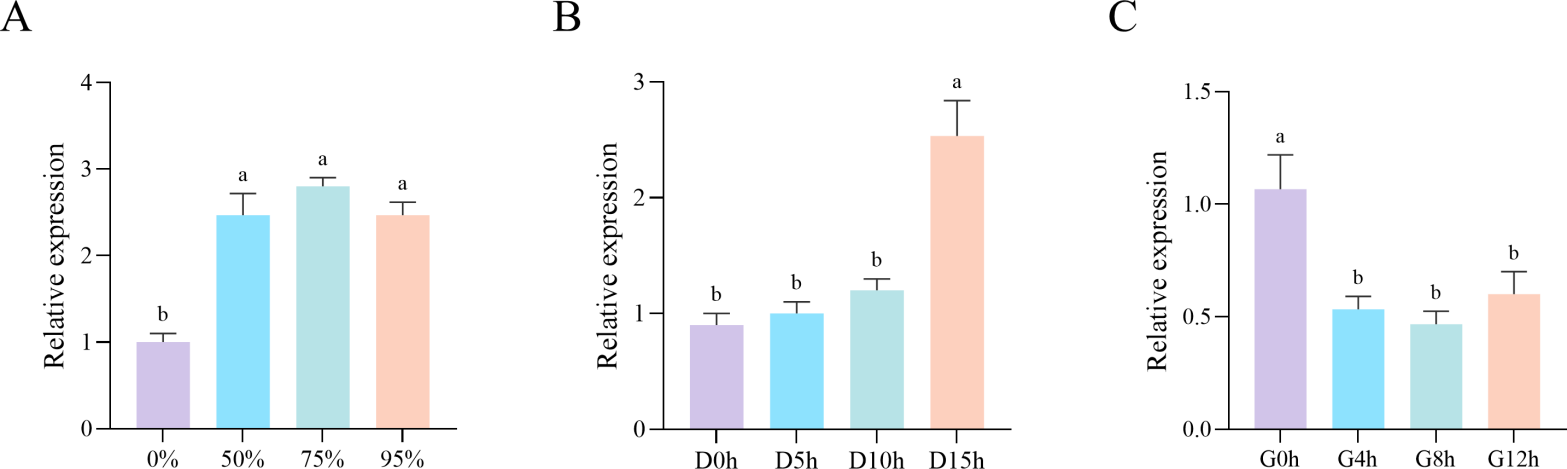
qPCR of *CmHDZ15* expression levels. **(A–C)** qPCR of the *CmHDZ15* expression levels in *Castanea mollissima* leaves under shade, cold, and heat stress, respectively.

Transcriptome data were downloaded from various databases and analyzed to understand the expression pattern of the *CmHDZ* genes under different stress treatments (**Figure 6**). The expression of *CmHDZ11* was not detected, whereas the other *CmHDZ* genes were differentially expressed under different conditions. Compared with the gene expression levels of the HDZ II and III members, those of the HDZ I and IV subfamilies were higher. Most of the *CmHDZ* genes (e.g., *CmHDZ2* and *CmHDZ8*) had very low expression levels under the four stress conditions (viz., shading, low or high temperatures, and drought). Only a few genes (e.g., *CmHDZ5* and *CmHDZ15*) were relatively highly expressed in response to stress. Notably, *CmHDZ15* showed very obvious changes in expression levels under three treatment conditions (shading and low- and high-temperatures). The transcriptome-based expression patterns of *CmHDZ15* were validated using the qPCR assay, whereupon the expression trends were found to be consistent. Under shading stress, the expression level of *CmHDZ15* first increased and then decreased; under low-temperature treatment, its expression level gradually increased; and under high-temperature treatment, its expression level first decreased and then increased. These results confirmed the credibility of the transcriptome data (**Figure 7C**).

### 3.8 Subcellular localization of CmHDZ15

**Figure 8:**
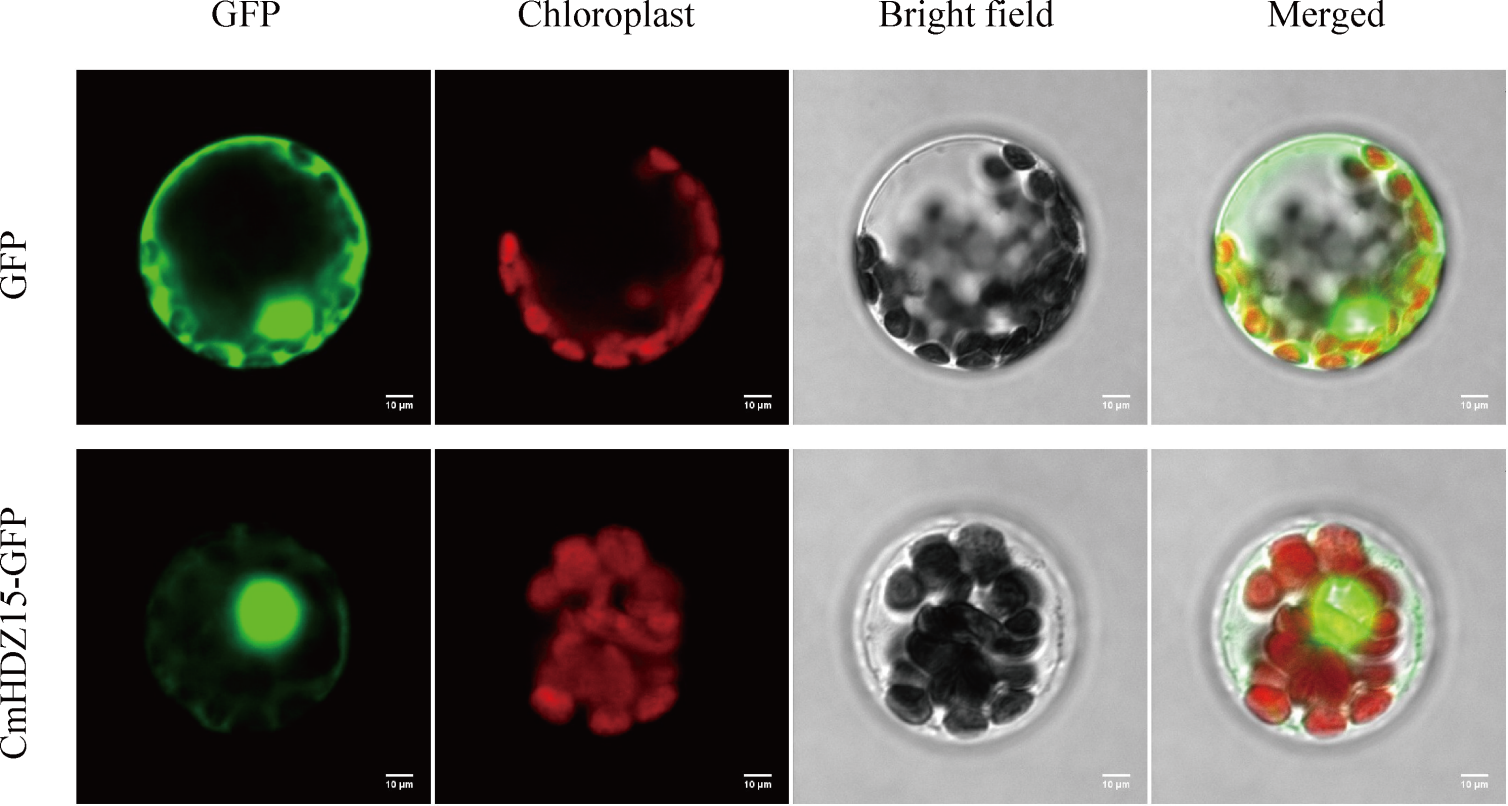
Subcellular localization of the CmHDZ15 protein.

The subcellular localization of proteins is closely related to their function. CmHDZ15 was selected for subcellular localization analysis because it showed the most obvious expression level changes in response to various stressors. The results showed that the CmHDZ15-GFP fusion protein was localized to the nucleus (**Figure 8**), indicating that CmHDZ15 may play the role of a transcription factor under different stress conditions.

## 4 Discussion

The growth and development of plants and their adaptation to the environment are regulated by several transcription factors. The HDZ members are a family of plant-specific transcription factors that have been widely studied in *Arabidopsis* (48 members), *O. sativa* (49 members) (Jain et al., 2008), *V. vinifera* (33 members) (Li et al., 2017), soybean (*Glycine max*: 88 members) (Belamkar et al., 2014; Chen et al., 2014), cassava (*Manihot esculenta*: 57 members) (Ding et al., 2017), wheat (*Triticum aestivum* L.: 46 members) (Yue et al., 2018), tea tree (*Camellia sinensis*: 33 members) (Shen et al., 2019), and potato (*Solanum tuberosum* L.: 43 members) (Li et al., 2019). Compared with these plants, *C. mollissima* had the fewest *CmHDZ* genes, with only 25 members identified in its genome. No definite correlation between the number of family members and the genome size has been found among different species, suggesting that the *CmHDZ* gene family has undergone different degrees of expansion during the evolution of different species. Moreover, studies have shown that the expansion of gene families mainly depends on tandem, segmental, and whole-genome duplications (Xu et al., 2012). Based on the analysis of *HDZ* gene family members in multiple species, this phenomenon may be attributed to the different frequencies of segmental and tandem duplications of plant *HDZ* family genes during the evolution of different species. Gene duplication and collinearity analyses have revealed that segmental duplication is the main driving force for *CmHDZ* gene expansion. Genome duplication is a fundamental force in the origin and evolution of species. However, during the evolutionary process, most of these duplications have disappeared or become silent, and the remaining few play important roles in positive or purifying selection (Lynch and Conery, 2000). The nonsynonymous/synonymous substitution rate (Ka/Ks value), an indicator of selection pressure, is commonly used to study the evolutionary direction and selection intensity of protein-coding genes. Ka/Ks values greater than 1, equal to 1, and less than 1 indicate positive, neutral, and purifying selection, respectively. In this study, eight pairs of gene duplication events were identified among the 25 *CmHDZ* genes, and their Ka/Ks values were all less than 1, indicating that these duplicated genes were subject to severe purifying selection (Yang et al., 2006).

Phylogenetic analysis of genes is beneficial for predicting their functions in a given plant species. The genes in the *HDZ* family are classified into four subfamilies on the basis of their structures and functions, with the number of members in each varying across different species. For example, *A. thaliana* contains 17, 9, 5, and 16 gene members in HDZ I, II, III, and IV, respectively (Mukherjee et al., 2009), whereas mulberry (*Morus alba*) possesses 14, 8, 3, and 8 members in the respective subfamilies. By contrast, *C. mollissima* possesses 13, 7, 4, and 1 members in the respective subfamilies. Large numbers of gene losses occurred during the whole-genome duplication process in *C. mollissima*.

According to the functional classification of the four subfamilies, members of HDZ I and II are involved in plant stress (Olsson et al., 2004) and auxin-mediated phototropic responses (He et al., 2020). Purportedly, the *CmHDZ* family members may be the result of long-term co-evolution with the environment. Transcription factors play a crucial role in the transcriptional regulation of plant stress responses, and their spatiotemporal expression patterns are regulated by *cis*-acting elements in their gene promoter regions. The *cis*-acting elements of the *CmHDZ* genes mainly included hormone response, light signal response, and stress response elements, which are similar to those of *HDZ* genes in species such as upland cotton (*Gossypium hirsutum*) and naked oat (*Hordeum vulgare* var. *nudum*), suggesting that these genes may be functionally conserved among different plant species. The HDZ I genes in *Arabidopsis* have been shown to be regulated by drought, high temperatures, and light (Olsson et al., 2004). The *CmHDZ* genes also contained stress response-related elements, suggesting that their expression may be regulated by light and temperature.

The species-level collinearity analysis indicated that the *HDZ* genes of *C. mollissima* and *A. thaliana* have a closer homologous evolutionary relationship than that between *C. mollissima* and *O. sativa*, *V. vinifera*, and *Z. mays*. The heatmap also showed that most of the HDZ I and II members only had collinearity with those of dicotyledonous plants, suggesting that these CmHDZ members formed after the differentiation of monocotyledonous and dicotyledonous plants. *CmHDZ8*, *CmHDZ15*, and *CmHDZ24* had collinear members in all species examined, suggesting that they may play an important role in plant adaptation.

The tissue-specific expression patterns indicated that the 25 *CmHDZ* genes were differentially expressed in various plant parts. Compared with the genes in the HDZ II and III subfamilies, those in HDZ I and IV were more highly expressed under stress conditions. Notably, the *CmHDZ15* expression levels changed markedly under three different stress treatments, indicating that this gene may exhibit functional diversity in the growth and development of *C. mollissima* and in response to adverse conditions, which is consistent with the reported functional diversity of *HDZ* gene families. Several studies have shown that plant *HDZ* genes are involved in responses to various abiotic stressors, such as in wheat (Yue et al., 2018), potato (Li et al., 2019), tea tree (Shen et al., 2019), and sesame (Wei et al., 2019). However, no reports exist on the responses of *CmHDZ* genes to cold, heat, shading, and drought. Therefore, this study is the first to systematically analyze the expression patterns of the *CmHDZ* genes under these four stress conditions. According to the results of the transcriptome data analysis, the expression levels of approximately 40% of the *CmHDZ* family members are affected by cold, heat, shading, and drought stress. Notably, the expression levels of *CmHDZ15* underwent significant changes under three of these treatments. Additionally, *CmHDZ15* was found to be closely related to *AtHB5*, *AtHB6*, and *AtHB16*. In this respect, *AtHB6* in *Arabidopsis* has been found to be expressed in developing leaves, roots, and carpels and is significantly upregulated in seedlings subjected to water deficit, osmotic stress, or exogenous ABA treatment, indicating that this gene is related to stress responses. These findings suggest that *CmHDZ15* may also be involved in the plant response to abiotic stress.

qPCR analysis confirmed that *CmHDZ15* is involved in abiotic stress responses and has different expression patterns depending on the type of stress encountered. Moreover, the CmHDZ15 protein is located in the nucleus and may function as a transcription factor that exerts regulatory effects. However, its stress resistance function should be verified via its heterologous transformation in *Arabidopsis*. Nonetheless, this study provides reliable evidence for the participation of *HDZ* genes in the abiotic stress responses of *C. mollissima* and lays a solid foundation for the future functional research of related genes.

After completing the evolutionary analysis of the members of this gene family, we will use genetic transformation techniques to analyze their heterologous expression in model plants such as *A. thaliana* or *Nicotiana tabacum* to further explore their biological functions. Subsequently, by analyzing the phenotypic and physiological biochemical changes of the transgenic plants under various abiotic stress conditions (drought, salt, low temperature, etc.), we will clarify the role of these genes in the stress resistance mechanisms of *C. mollissima*.

## Data Availability

Publicly available datasets were analyzed in this study. This data can be found here: https://ngdc.cncb.ac.cn/gsa.
